# The Hyponatremia Epidemic: A Frontier Too Far?

**DOI:** 10.3389/fcvm.2016.00035

**Published:** 2016-10-07

**Authors:** Angela J. Drake-Holland, Mark I. M. Noble

**Affiliations:** ^1^School of Pharmacy and Life Sciences, The Robert Gordon University, Aberdeen, UK; ^2^Department of Medicine and Therapeutics, University of Aberdeen, Aberdeen, UK

**Keywords:** sodium, salt, elderly, kidney function, hypertension

## Abstract

Hyponatremia is the most common electrolyte abnormality and is often neglected, especially in elderly and seemingly terminal patients. Hyponatremia can be asymptomatic or can cause symptoms ranging from nausea and lethargy to convulsions and coma. This condition has become increasingly common over time with a similar time course to the increase in adoption of low salt diets. The popularization of low salt may not be justified in people with normal kidney function in whom the compatible statistically based evidence that salt causes hypertension has been challenged by experimental evidence to the contrary.

## Key Points

1. Hyponatremia is the most common electrolyte abnormality and is often neglected, especially in elderly and seemingly terminal patients.
2. Hyponatremia can be asymptomatic or can cause symptoms ranging from nausea and lethargy to convulsions and coma.
3. The increase in prevalence over time has a similar time course to the increase in adoption of low-salt diets.
4. Investigation into the electrolyte status of individual subjects would be expected to reveal the suitability or otherwise of dietary salt intake.
5. If policy 4 above is adopted, it may be necessary to withdraw recommendation of low-salt diet to the entire population.
6. Investigation into the electrolyte status of individual subjects would also be advisable in view of the lack of experimental evidence in normal people that high salt raises blood pressure and that hypertensive patients have normal body sodium.

## Hyponatremia

A normal concentration of sodium ions (Na^+^) in plasma and extracellular fluid is essential for nerve and muscle electrical conduction (1, 2). If dietary salt intake does not equal or exceed salt loss in urine and sweat, a fall in Na^+^ below concentrations that allow normal function will occur (2). This condition (hyponatremia) causes dysfunction of brain and body; much Alzheimer’s disease has been attributed to hyponatremia (3). In a hot country, such as Uganda, in which there is a high sweating rate that loses salt, the prevalence of hyponatremia in hospitalized patients over the age of 60 was as high as 25% (4). Hyponatremia is also associated with diuretic treatment (5, 6).

The prevalence of hyponatremia should highlight the magnitude of the problem and should prompt health-care professionals to investigate and treat these patients and terminate low salt diet if being adopted by elderly and chronic sick individuals to their detriment. Low salt diet is associated with increased mortality and has been heavily criticized in the US press and the Lancet (7). As long as sodium excretion is normal, a high salt diet is safe; if such excretion is excessive, as is often the case in the elderly, low salt diet is very dangerous. In subjects with normal or increase Na^+^ excretion, an increase salt intake could improve the quality of life.

Squecco et al. (8) have recently reported detrimental effects of hyponatremia on two different neuronal cell lines, SK-N-AS and SH-SY5Y, independent of osmotic alterations. They made experiments in low sodium medium either in hypo-, hyper-, or iso-osmotic conditions, for a short (24 h) or long time (7 days). Their results indicate that cell capacitance and membrane conductance increase regardless of osmolarity, but such effects are differently dependent on time in culture. Relevant to the mechanisms involved in maintenance of cell capacitance and membrane conductance in hyper-osmotic medium, they also observed a greater contribution of the sodium–calcium exchanger with respect to hypo-osmotic medium and control. In view of these fundamental changes in basic neuronal electrophysiology induced by hyponatremia, and the basic fact that depolarization is a sodium-dependent function in electrically active cells, e.g., heart, vascular smooth muscle, and skeletal muscle, we can only emphasize the need to avoid hyponatremia at all cost.

## The Increasing Prevalence

There are an increasing number of reports of the rising prevalence of hyponatremia, e.g., Nair et al. (9), Lobo-Rodríguez et al. (10), and Gandhi et al. (11) even in children (who surely should not be given a low salt diet) (12), including neonates (13) and athletes who drink too much water when losing salt through sweating (14, 15). Pituitary deficiency of ACTH (16), subarachnoid hemorrhage (17), osteoporosis, failure of salt supplementation with medical treatments (18), thiazide treatment in the elderly (19), and thiazide and psychotropic drugs combined (20) have all been associated with severe hyponatremia. When these conditions occur in a population on low salt intake, a considerable possible risk may result in unnecessary morbidity, mortality, and strain on health services.

## The State Advice to Populations to Adopt the Low Salt Diet, i.e., Should Normal People Eat a Low Salt Diet?

Many states advise their populace to eat a low salt diet, an advice that is based on the many studies, indicating that this will save lives (21–27). We protest about the spurious argument that salt intake has increased in recent times (28). The historical evidence is quite opposite, as early civilizations, the Navy, and housewives have until recently used salt for preserving food. The most precious gift a guest could offer his host in previous civilizations was “bread and salt.” Now, with food being preserved in freezers, salt intake has decreased. The debate continues (28, 29).

Meta-analyses of trials in normotensive subjects are similar to prospective studies in estimating a 1.7 mmHg rise per 100-mM increment in urinary 24-h sodium excretion, but prospective studies relating *health outcomes* to 24-h sodium excretion produced inconsistent results (30–32). The fall in BP with salt restriction may be greater in patients with diabetic kidney disease than in normals (33). In a very large study in European White ethnic subjects, in whom sodium intake was accurately assessed by measuring 24-h urinary sodium output, no correlation between salt intake and blood pressure was shown (34), suggesting that the earlier studies on mixed ethnic populations were unsound, as salt sensitivity varies in various populations (2). When normal people with normal renal function ingest sodium, any excess is excreted in the urine, and total body sodium remains normal (35). The issue of whether nations should be on low salt could be decided by a double-blind controlled clinical trial of similar groups of normal people randomized to low-salt versus high-salt diet and followed up for a lifetime. The end points would be age at death, clinical hypertension confirmed by automated 24-h blood pressure monitoring, and plasma sodium ion concentration.

In view of the personal and economic burden on health provision of hyponatremia, a wiser policy in the meantime might be for any normotensive person wondering whether to restrict their salt intake to undergo renal function tests and a salt loading test. If there is no contraindication, a normal salt diet could be prescribed with the proviso that the tests be repeated if the patient develops hypertension confirmed by automated 24-h blood pressure monitoring.

## Direct Experimental Evidence Pertaining to Salt Ingestion and Body Sodium

### Salt Loading

We (36) made a statistical analysis of the salt loading study of Luft et al. (37). The relationship, of systolic BP to salt loading of 7 days, in that study showed no significance (Spearman rank correlation), until doses of over 50 g/day (Caucasian subjects, *n* = 7). The other group of Afro-Caribbean subjects (*n* = 7) started to show a small increase in BP at 20 g/day, showing that ethnicity is important. Studies with longer periods of salt-loading and more subjects [4 weeks (38), 9 weeks (39)] found no increase in BP in normotensive and hypertensive subjects, respectively. Experimentally, high salt ingestion has no effect on blood pressure in 9 weeks, but there remains the possibility that salt loading for longer durations might cause a rise in BP.

### Total Exchangeable Sodium

The Medical Research Council Blood Pressure Group in Glasgow measured total exchangeable sodium in normals, patients developing hypertension and patients with established hypertension (40). There were no differences between the three groups. Hypertension is not associated with increased body sodium.

### Dietary Salt Reduction

In order to test the idea that salt restriction would lower blood pressure, Sacks et al. (41) performed a thorough study in normotensive subjects of the effect of salt restriction and found decreases of average systolic blood pressure between 2.2 and 5.9 mmHg and decreases of average diastolic blood pressure between 1.0 and 2.9 mmHg.

## Should Salt Restriction be Administered to Hypertensive Patients?

In some non-Caucasian ethnic groups, the so-called “low renin-hypertension” is an important, and sometimes overlooked, cause. This needs to be managed for each individual as there may be a logical decision to impose a low salt diet. The same result of the individual consultation process may be implemented in patients whose hypertension has a definable cause such as kidney disease. This situation highlights the need for people’s health or health problems to be supervised for them as individuals. The great Nobel Prize laureate, Feynman (42), deprecates politicians making generally applicable policies based on only one side of a scientific argument and not necessarily applicable to all individuals.

The commonist underlying metabolic abnormality in hypertensive patients is insulin resistance or “metabolic syndrome,” frequently accompanied by obesity. In these patients, high blood pressure responds satisfactorily to weight reduction (43). It can safely be assumed that weight reduction, particularly carbohydrate intake reduction, is also an effective treatment for the insulin resistance. There is evidence to suggest that BP is lowered in hypertension by Mediterranean and Nordic diets (44); so it will be extremely difficult to separate the effect of low salt from all the other ingredients of diet that may lower BP.

It is unusual for salt restriction to be necessary in the treatment of clinical hypertension. In the typical patient in Europe, the key to clinical management is the relationship between sodium and the renin–angiotensin system (45, 46). In these patients, who are the majority, suppression of the activity of the renin–angiotensin system by angiotensin-converting enzyme (ACE), or angiotensin II receptor antagonism, is effective in both controlling the BP and reducing the complications of hypertension. Diabetes also occurs in the insulin resistance triad of obesity/hypertension/diabetes, but salt restriction makes diabetes worse (2). It is illogical for such patients to also indulge in salt restriction, unless they have kidney disease, so doctors do not recommend it. Perhaps salt restriction not supervised for the individual by his doctor contributes to the increasing prevalence of hyponatremia in patients with heart failure, the commonist end condition of uncontrolled hypertension?

## Which Populations Should Receive High-Salt and Which Low-Salt Diets?

1. Patients with low exchangeable sodium values should have a high rather than a low-salt diet, while patients with specified types of cardiovascular disease such as low-renin hypertension should be considered for low-salt diet.
2. The consequence of low salt in elderly and chronic sick can reasonably be postulated to cause increased severity of illness. A possible additional hypothesis is that low-salt diet in the not obviously ill person may induce hyponatremic illness.
3. Of particular concern in addition is the ingestion of inadequate salt intake without adequate investigation in hot climates and patients with severe cardiovascular disease and cancer.

## Conclusion

The increasing prevalence of hyponatremia is a worrying development and an increasing burden for health services. Whether this increasing prevalence is related in any way to the increasing adoption by the population of low salt diet cannot be proved, only postulated. The relationship between salt intake and hypertension also cannot be proved, only postulated, because there is contrary evidence and a continuing debate. Low-salt diet is only recommended as treatment of hypertension in single individuals following thorough renal and endocrinological investigations. The possibility that universal low salt ingestion by the population might perhaps contribute to the prevalence of hyponatremia should be sufficient reason for such universal ingestion to be replaced by individual care of individuals by the individual’s doctor.

## Author Contributions

MN created the basic outline, read references, discussions of content, and wrote 50% of the manuscript. AD-H researched references in Pubmed, created table, wrote 50% of manuscript, and edited the manuscript.

## Conflict of Interest Statement

The authors have not received payment or services of any third party for any aspect of the submitted work and have no financial relationships with entities that could be perceived to influence or that give the appearance of potentially influencing what has been written in the submitted work. The authors have no declared or pending patents, copyrights, or royalties relevant to the work. The authors have no other relationships or activities that could be perceived as having influenced or potentially influencing what has been written in the submitted work.

## References

[1] Mitchell AR Physiological aspects of the requirement for sodium in mammals. Nutr Res Rev (1989) 2:149–60. 10.1079/NRR19890012 19094351

[2] Gupta N Jani KK Gupta N. Hypertension: salt restriction, sodium homeostasis, and other ions. Indian J Med Sci (2011) 65:121–32. 10.4103/0019-5359.104787 23250294

[3] Alzheimer Research Forum Live Discussion: sports concussions, dementia, and ApoE genotyping: what can scientists tell the public? What’s up for research? J Alzheimers Dis (2009) 16(3):657–66. 10.3233/JAD-2009-1014 19276560

[4] Nankabirwa H Kalyesubula R Ssinabulya I Katabira ET Cumming RG. A cross-sectional study of hyponatraemia among elderly patients with heart failure in Uganda. BMJ Open (2016) 6:5. 10.1136/bmjopen-2015-009775 27188802 PMC4874129

[5] Sonnenblick M Friedlander Y Rosin AJ. Diuretic-induced severe hyponatremia. Review and analysis of 129 reported patients. Chest (1993) 103:601–6. 10.1378/chest.103.2.601 8432162

[6] Hix JK Silver S Sterns RH Diuretic-associated hyponatraemia. Semin Nephrol (2011) 31:553–66. 10.1016/j.semnephrol.2011.09.010 22099512

[7] Editorial: salt and cardiovascular disease mortality. Lancet (2011) 317:1626. 10.1016/S0140-6736(11)60657-0 21571130

[8] Squecco R Luciani P Idrizaj E Deledda C Benvenuti S Giuliani C Hyponatremia alters the biophysical properties of neuronal cells independently of osmolarity: a study on Ni^2+^-sensitive current involvement. Exp Physiol (2016) 101(8):1086–100. 10.1113/EP085806 27307205

[9] Nair S Mary TR Tarey SD Daniel SP Austine J. Prevalence of hyponatremia in palliative care patients. Indian J Palliat Care (2016) 22:33–7. 10.4103/0973-1075.173954 26962278 PMC4768447

[10] Lobo-Rodríguez C García-Pozo AM Gadea-Cedenilla C Moro-Tejedor MN Pedraz Marcos A Tejedor-Jorge A Prevalence of hyponatraemia in patients over the age of 65 who have an in-hospital fall. Nefrologia (2016) 36(3):292–8. 27161308 10.1016/j.nefro.2016.03.014

[11] Gandhi S McArthur E Reiss JP Mamdani MM Hackam DG Weir MA Atypical antipsychotic medications and hyponatremia in older adults: a population-based cohort study. Can J Kidney Health Dis (2016) 3:21. 10.1186/s40697-016-0111-z 27069639 PMC4827184

[12] Al Shibli A Abukhater D Al Kuwaiti N Noureddin MB Al Harbi M Al Kaabi A Hyponatraemia and neurological complications in children admitted with bronchiolitis. Paediatr Int Child Health (2016) 36(3):175–80. 10.1080/20469047.2016.1162390 27079908

[13] Tseng MH Lin SH Chung HT Hsu TC Lien R Huang JL Severe hyponatremia secondary to peripherally inserted central catheter in a neonate. Pediatr Neonatol (2016). 10.1016/j.pedneo.2015.08.010 27157406

[14] Maughan RJ Shirreffs SM. Dehydration and rehydration in competitive sport. Scan J Med Sci Sports (2010) 20(Suppl 3):40–7. 10.1111/j.1600-0838.2010.01207.x 21029189

[15] Valentine V The importance of salt in the athlete’s diet. Curr Sports Med Rep (2007) 6(4):237–40. 10.1097/01.CSMR.0000306477.87713.74 17617999

[16] Doroftei NA de Rudder C de Visscher N Hanon F. Isolated ACTH deficiency in a patient with empty sella as revealed by severe hyponatremia. Acta Clin Belg (2016):1–4. 10.1080/17843286.2016.1175702 27176077

[17] Mistry AM Mistry EA Ganesh Kumar N Froehler MT Fusco MR Chitale RV Corticosteroids in the management of hyponatremia, hypovolemia, and vasospasm in subarachnoid hemorrhage: a meta-analysis. Cerebrovasc Dis (2016) 42(3–4):263–71. 10.1159/000446251 27173669

[18] Polák P Husa P Kubešová HM Severe osteoporosis – the story of chronic medication-related hyponatremia. [Article in Czech]. Vnitr Lek (2016) 62(2):152–6. 27172443

[19] Liamis G Filippatos TD Elisaf MS. Thiazide-associated hyponatremia in the elderly: what the clinician needs to know. J Geriatr Cardiol (2016) 13(2):175–82. 10.11909/j.issn.1671-5411.2016.02.001 27168745 PMC4854958

[20] Yoon HJ Lee KY Sun IO. Clinical severity of drug-induced hyponatremia: thiazides vs. psychotropics. Clin Nephrol (2016) 85(6):321–5. 10.5414/CN108765 27125625

[21] Mohan S Campbell NR Salt and high blood pressure. Clin Sci (2009) 117:1–11. 10.1042/CS20080207 19476440

[22] He FJ MacGregor GA. Reducing population salt intake worldwide; from evidence to implementation. Prog Cardiovasc Dis (2010) 52:363–82. 10.1016/j.pcad.2009.12.006 20226955

[23] NICE. Cutting Salt and Fat Levels in Food Could Save Thousands of Lives. National Institute for Health and Clinical Excellence (2011). (Press release June 25, 2010); 07/02 10:54. Available from: http://www.nice.org.uk/newsroom/news/CuttingSaltAandFatLevels.jsp

[24] Institute of Medicine. Strategies to Reduce Sodium Intake in the United States – Consensus Report. Institute of Medicine of the National Academies (2011). (Press release April 20); 07/02 10:56. Available from: https://www.ncbi.nlm.nih.gov/books/NBK50956/

[25] Bibbins-Domingo K Chertow GM Coxson PG Moran A Lightwood JM Pletcher MJ Projected effect of dietary salt reductions on future cardiovascular disease. N Engl J Med (2010) 362(7):590–9. 10.1056/NEJMoa0907355 20089957 PMC3066566

[26] Ha SK Dietary salt intake and hypertension. Electrolyte Blood Press (2014) 1:7–18. 10.5049/EBP.2014.12.1.7 PMC4105387 25061468

[27] Webster J Trieu K Dunford E Nowson C Jolly KA Greenland R Salt reduction in Australia: from advocacy to action. Cardiovasc Diagn Ther (2015) 5:207–18. 10.3978/j.issn.2223-3652.2015.04.02 26090332 PMC4451308

[28] Batuman V Salt and hypertension: why is there still a debate? Kidney Int Suppl (2011) 4:316–20. 10.1038/kisup.2013.66 PMC4089608 25019011

[29] Simpson FO Salt and hypertension: a skeptical review of the evidence. Clin Sci (1979) 57:S463–80. 10.1042/cs057463s 396090

[30] Stolarz-Skrzypek K Liu Y Thijs L Kuznetsova T Czarnecka D Jaszcz K Blood pressure, cardiovascular outcomes and sodium intake, a critical review of the evidence. Acta Clin Belg (2012) 67:403–10. 23340145 10.2143/ACB.67.6.2062704

[31] Stolarz-Skrzypek K Bednarski A Czarmecka D Kawecka-Jaszcz K Staessen JA. Sodium and potassium and the pathogenesis of hypertension. Curr Hypertens Rep (2013) 15:122–30. 10.1007/s11906-013-0331-x 23397214

[32] Stolarz-Skrzypek K Staessen JA Reducing salt intake for prevention of cardiovascular disease – times are changing. Adv Chronic Kidney Dis (2015) 22:108–15. 10.1053/j.ackd.2014.12.002 25704347

[33] Suckling RJ He FJ MacGregor GA Altered dietary salt for preventing and treating diabetic kidney disease. Cochrane Database Syst Rev (2010) 8:12. 10.1002/14651858.CD006763.pub2 21154374

[34] Stolarz-Skrzypek K Kuznetsova T Thijs L Tikhonoff V Seidlerova J Richart T Fatal and nonfatal out-comes, incidence of hypertension, and blood pressure changes in relation to urinary sodium excretion. JAMA (2011) 305:1777–85. 10.1001/jama.2011.574 21540421

[35] Robertson JIS. Dietary salt and hypertension: a scientific issue or a matter of faith? J Eval Clin (2003) 9:1–22. 10.1046/j.1365-2753.2003.00359.x 12558698

[36] Drake-Holland AJ Noble MIM Commentary: should we now abandon the low salt diet? QJM (2011) 104:1103–6. 10.1093/qjmed/hcr124 21835780

[37] Luft FC Ramkin LI Bloch R Weyman AE Willis LR Murray RH Cardiovascular and humoral responses to extremes of sodium intake in normal black and white men. Circulation (1979) 60:697–706. 10.1161/01.CIR.60.3.697 455628

[38] Kirkendall AM Connor WE Abboud F Rastogi SP Anderson TA Fry M. The effect of dietary sodium chloride on blood pressure, body fluids electrolytes, renal function, and serum lipids of normotensive man. J Lab Clin Med (1976) 87:411–34. 1249473

[39] Gros G Weller JM Hoobler W Relationship of sodium and potassium intake to blood pressure. Am J Clin Nutr (1971) 24:605–8. 5580999 10.1093/ajcn/24.6.605

[40] Lever AF Beretta-Piccoli C Brown JJ Davies DL Fraser R Robertson JIS Sodium and potassium in essential hypertension. Br Med J (1981) 283:463–8. 10.1136/bmj.283.6289.463 6790017 PMC1506255

[41] Sacks FM Svetkey LP Vollmer WM Appel LJ Bray GA Harsha D Effects on blood pressure of reduced dietary sodium and the dietary approaches to stop hypertension (DASH) diet. N Engl J Med (2001) 344:3–10. 10.1056/NEJM200101043440101 11136953

[42] Feynman RP Surely You’re Joking Mr. Feynman. London: Vintage Books (1992).

[43] Reisin E Abel R Modan M Silverberg DS Eliahou HE Modan B Effect of weight loss without salt restriction on the reduction of blood pressure in overweight hypertensive subjects. N Engl J Med (1978) 298:1–6. 10.1056/NEJM197801052980101 337144

[44] Ndanuko RN Tapsell LC Charlton KE Batterham MJ Dietary patterns and blood pressure in adults: a systematic review and meta-analysis of randomised controlled trials. Adv Nutr (2016) 15:76–89. 10.3945/an.115.009753 PMC4717885 26773016

[45] Hall JE. Control of sodium excretion by angiotensin II: intrarenal mechanisms and blood pressure regulation. Am J Physiol (1986) 250:R960–72. 3521337 10.1152/ajpregu.1986.250.6.R960

[46] Hall JE Guyton AC Mizelle HL. Role of the renin-angiotensin system in control of sodium excretion and arterial pressure. Acta Physiol Scand Suppl (1990) 591:48–62. 2220409

